# Diagnostic and Therapeutic Nuances in Cerebral Amyloid Angiopathy–Related Inflammation: Case Reports and Literature Review

**DOI:** 10.1155/crnm/9463046

**Published:** 2026-11-15

**Authors:** Tal Zusman, Maria Muñoz, Maria V. Diaz, Lisa José, Marcos Mota, Rebecca Frawley, Chloe Dominguez, Nicole Heath, David Hammer, Aparna Prabhu, Fatima Alao, Ryna Then

**Affiliations:** ^1^ Department of Neuroscience Neurology Division Jefferson Einstein Philadelphia Hospital Philadelphia Pennsylvania USA; ^2^ Institute of Technology of Santo Domingo Santo Domingo Dominican Republic; ^3^ Sarah Bush Lincoln Hospital Mattoon Illinois USA

**Keywords:** β-amyloid, autoimmune, CAA-ri, case report

## Abstract

Cerebral amyloid angiopathy–related inflammation (CAA‐ri) is a rare autoimmune response to vascular *β*‐amyloid deposition that poses a significant diagnostic challenge. Its clinicoradiological criteria require evidence of cortical or subcortical microhemorrhage or superficial siderosis markers whose detection depends on the field strength and sequence of the study. We describe two cases that bracket this problem. In the first case, a 74‐year‐old woman presented with subacute lethargy and a right parietal lesion initially concerning for an infiltrating low‐grade glioma on 1.5 T MRI. Two gradient‐echo studies at 1.5 T 1 month apart showed no microhemorrhages, and she, therefore, did not satisfy the criteria for probably CAA‐ri. Subsequent ultra‐high‐field 7 T MRI demonstrated numerous cortical microhemorrhages not captured on conventional imaging, shifting the clinical suspicion toward CAA‐ri. Brain biopsy was performed confirming vascular amyloid involvement. The patient experienced spontaneous radiographic resolution without corticosteroid therapy. In the second case, an 84‐year‐old woman with a history of hypertension and dementia presented with progressive confusion. Neuroimaging revealed severe, confluent white matter hyperintensities and multiple microhemorrhages consistent with CAA‐ri, satisfying criteria for probably CAA‐ri on 1.5 T imaging alone. Unlike the first case, this patient received high‐dose intravenous methylprednisolone, resulting in rapid clinical stabilization and subsequent resolution of vasogenic edema on follow‐up imaging. Together, these cases demonstrate divergent diagnostic outcomes under identical criteria. One patient was identified within days on conventional imaging while the other underwent extensive neoplastic evaluation. The determinant was the detectability of the microhemorrhages on the acquired sequence rather than difference in disease phenotype. This limitation extends to screening for antiamyloid monoclonal antibody therapy in which eligibility is determined by microhemorrhage counts obtained on conventional MRI. Maintaining a high index of suspicion for CAA‐ri is essential when evaluating atypical intracranial lesions in older adults to ensure timely diagnosis and appropriate therapeutic management.

## 1. Introduction

Cerebral amyloid angiopathy–related inflammation (CAA‐ri) is a rare and poorly understood disorder caused by an inflammatory response to *β*‐amyloid deposition in cerebral blood vessels [1]. Clinically, it often presents as an acute or subacute course of rapidly progressive cognitive decline and behavioral changes. Patients may also develop seizures and focal neurological deficits, including weakness, speech disturbances, or visual impairments [2]. Radiologically, CAA‐ri can closely mimic low‐grade gliomas, posing significant diagnostic challenges [3]. Moreover, the clinical and imaging features may overlap with other conditions such as primary angiitis of the central nervous system, demyelinating diseases, or infections, further complicating the diagnostic process [1]. Confirmation therefore relies on a combination of clinical suspicion, neuroimaging findings, and, in selected cases, histopathological evaluation through brain biopsy [1].

Brain magnetic resonance imaging (MRI) of CAA‐ri is characterized by asymmetric white matter hyperintensities that extend into the subcortical regions, associated with cerebral microbleeds or cortical superficial siderosis, and occasionally by leptomeningeal enhancement after contrast administration [4]. Cerebrospinal fluid may show evidence of inflammation [5], including mild lymphocytic pleocytosis and increased protein, although these findings are not universally present. Interestingly, acute episodes of the disease have been linked to the presence of anti–amyloid‐β autoantibodies in CSF, reinforcing the concept of an underlying immune‐mediated reaction to vascular amyloid deposition in the brain [6]. Management of CAA‐ri is mainly based on immunosuppressive therapy, with corticosteroids being the cornerstone of treatment. Standard regimens usually begin with high‐dose intravenous steroids, followed by a gradual tapering schedule that often extends over several months [7].

While the established diagnostic criteria provide a framework for identifying CAA‐ri, the variable nature of its presentation ensures that it remains a significant diagnostic challenge in clinical practice. Overlap with neoplastic and inflammatory mimics often leads to invasive procedures or delays in starting medication. A less examined source of difficulty is that the criteria presuppose hemorrhagic markers are visible on the imaging obtained. Detection of cortical microbleeds improves substantially at higher field strength and the criteria are not validated at 7 T, so the patient may fall outside criteria on one study and inside on another without change in underlying disease. The following case studies illustrate the diverse spectrum of CAA‐ri, highlighting the critical role of advanced neuroimaging and the rapid clinical stabilization achievable with targeted corticosteroid therapy. We present them as a deliberate pair. In the first patient, the imaging markers required for diagnosis were clearly visible on standard MRI. In the second, the same markers could not be seen until ultra‐high‐field MRI was performed. By synthesizing these findings with a comprehensive literature review, we aim to clarify the diagnostic nuances and therapeutic trajectories that define this complex neuroinflammatory entity.

## 2. Case Reports

### 2.1. Case 1

A 74‐year‐old woman with hypertension, heart failure, monoclonal gammopathy of undetermined significance, Raynaud’s phenomenon, anxiety disorder, migraines, restless leg syndrome, and chronic hyponatremia was referred to the emergency department for acute on chronic hyponatremia and acute kidney injury.

On examination, the patient was alert but lethargic, with no focal neurological deficits. Noncontrast head computed tomography (CT) revealed a right posterior parietal hypodensity. Brain MRI at 1.5 T showed focal vasogenic edema in the same region, without contrast enhancement and without evidence of hemorrhage on gradient‐echo (GRE) images. The findings were initially suggestive of a low‐grade tumor versus cerebritis with negative malignancy workup. Electroencephalography (EEG) showed no epileptiform activity. She was treated empirically with dexamethasone 4 mg every 6 h and improved to baseline mental status. Steroids were discontinued shortly after initiation due to patient preference.

Repeat 1.5 T MRI after 1 month demonstrated slight progression of cortical thickening in the right temporo‐occipital region, no enhancement or GRE abnormalities, favoring a diagnosis of low‐grade glioma (Figure 1A,B) without microhemorrhages (Figure 1C). Because of the interval change, a 7 T MRI with and without contrast was performed, showing further enlargement of the FLAIR hyperintense lesion (Figure 2A,B), but now revealing numerous cortical microhemorrhages on GRE (Figure 2C). The differential diagnosis included low‐grade glioma and inflammatory cerebral amyloid angiopathy. Magnetic resonance spectroscopy showed no increase in choline or hyperperfusion, suggesting low likelihood of tumoral process.

**FIGURE 1:**
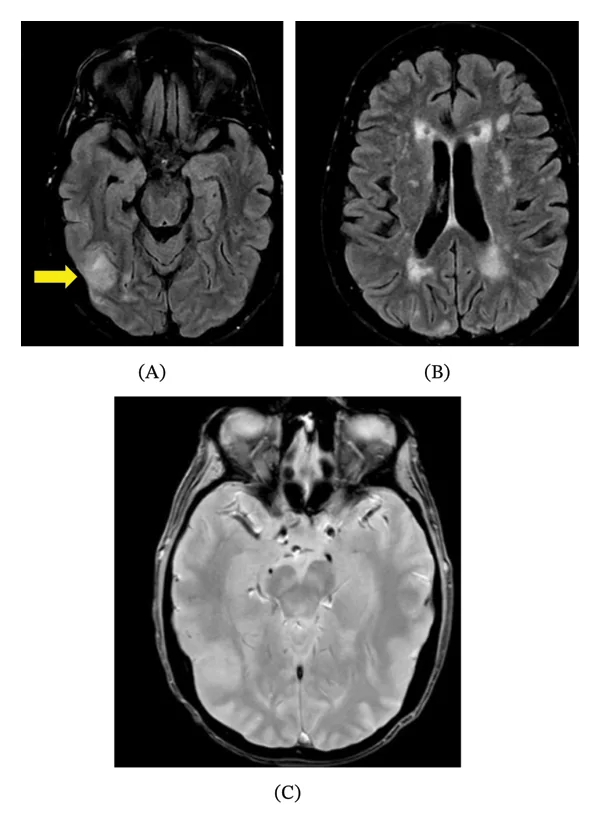
Brain MRI, 1.5 T (FLAIR sequence) showed (A) focal right posterior temporal hyperintensity and cortical thickening and (B) multifocal white matter ischemic disease. Brain MRI, 1.5 T (GRE sequence) showed (C) no evidence of microhemorrhages.

**FIGURE 2:**
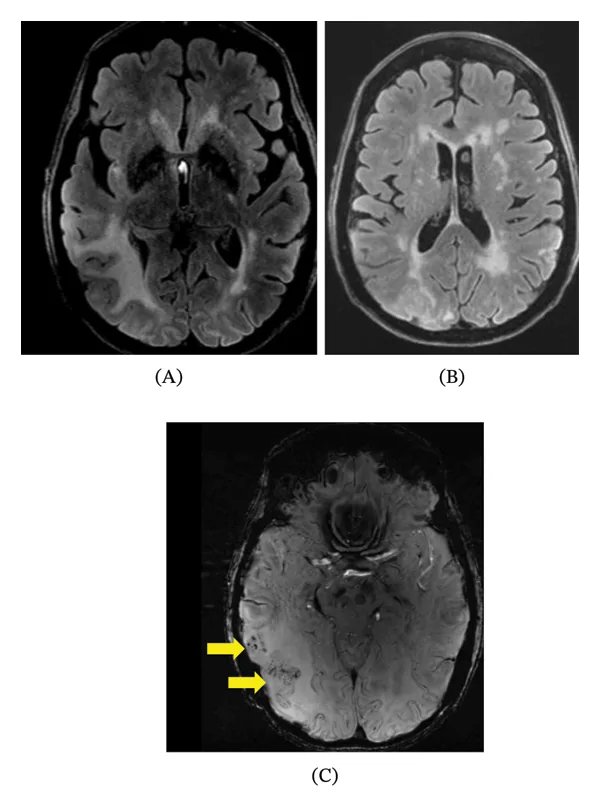
Brain MRI, 7 T (FLAIR sequence) showed (A) enlargement of right posterior temporal hyperintensity and cortical thickening and (B) worsening multifocal white matter ischemic disease. Brain MRI, 7 T (GRE sequence) showed (C) evidence of right temporal microhemorrhages.

Twenty days later, the patient presented to the hospital with two generalized tonic‐clonic seizures. Long‐term EEG monitoring demonstrated right‐hemispheric lateralized epileptiform discharges, and she was started on levetiracetam 1000 mg twice daily. A repeat MRI showed a mild interval increase in the same lesion with additional microhemorrhages. A stereotactic brain biopsy was performed, and histopathological analysis revealed mildly increased cellularity and no evidence of malignancy. Immunohistochemistry showed widespread amyloid deposition in vessel walls and numerous amyloid plaques throughout the cortex, confirming the diagnosis of cerebral amyloid angiopathy. Genetic and molecular testing was normal.

The patient chose not to receive high‐dose steroids. Serial MRIs were performed every 3 months showing gradual resolution of the previously noted edema and abnormal signal, with near‐complete resolution after 6 months. At her last follow‐up, she remained seizure‐free on levetiracetam, with mild cognitive impairment and no motor deficits.

### 2.2. Case 2

An 84‐year‐old woman with a history of hypertension, diabetes mellitus type 2, dementia, and paroxysmal atrial fibrillation (not on anticoagulation) presented with altered mental status and uncontrolled hypertension. Her caregiver reported progressive confusion, wandering behavior, and disorientation over the preceding months.

On examination, she was alert and oriented to person and place without focal neurological deficits. A noncontrast head CT revealed new left frontal and right frontoparietal hypodensities in the subcortical white matter, small vessel disease, and cerebral atrophy. CT angiography showed occlusion of a hypoplastic left vertebral artery at the V3–V4 junction, no evidence of large‐vessel occlusion. MRI demonstrated severe white matter hyperintensities and multiple cortical microhemorrhages (Figure 3A–D). Applying the Auriel criteria [8], she met every element for probable CAA‐ri on 1.5 T imaging alone. She was older than 40 years and had subacute behavioral changes with progressive confusion, wandering, and disorientation over the preceding months. Imaging showed white matter hyperintensity extending into the subcortical white matter and multiple cortical subcortical microhemorrhages on GRE sequences. No neoplastic, infectious, or alternative vascular explanation was identified, and there was no large vessel occlusion. A diagnosis of probable CAA‐ri was, therefore, made without biopsy.

**FIGURE 3:**
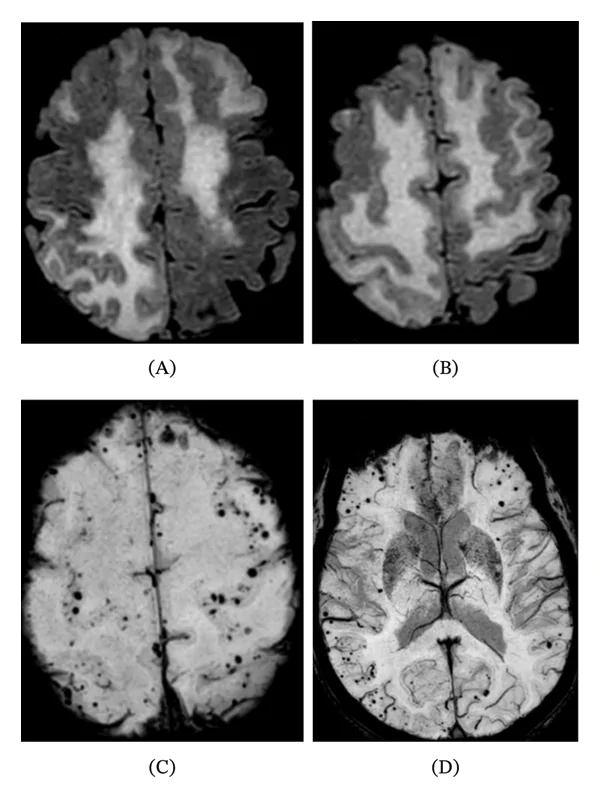
Brain MRI, 1.5 T (FLAIR sequence) showed (A, B) extensive vasogenic edema involving frontal and parietal white matter with sulcal effacement. Brain MRI, 1.5 T (GRE sequence) showed (C, D) evidence of multiple cortical and subcortical microhemorrhages.

The patient was treated with intravenous methylprednisolone 1 g daily for 5 days, followed by oral prednisone 60 mg daily with a gradual taper planned for over 3 months. During hospitalization, she showed mild improvement in attention and alertness. Genetic testing for apolipoprotein E4 (ApoE4) and follow‐up MRI were recommended to evaluate Alzheimer’s pathology, but these were not completed due to lack of follow‐up.

Eight months later, she presented to the emergency department after being found wandering the streets. She remained pleasantly confused and oriented only to person and place, with a blood pressure of 179/83 mmHg and no focal neurological deficits. Repeat head CT demonstrated resolution of the previously noted vasogenic edema.

## 3. Discussion

The cases presented illustrate the distinct clinical and radiographic manifestation of CAA‐ri and highlight the diagnostic challenges that arise when various differentials present with overlapping characteristics. Neurologists rely heavily on neuroimaging to confirm lesion localization and establish a differential diagnosis in most neurologic conditions, with MRI playing a central role in this process. Inflammatory, neoplastic, vascular, and infectious etiologies frequently share overlapping imaging findings, making accurate interpretation essential [9].

In Case 1, radiographic mimicry of a neoplastic process, a phenomenon well described in CAA‐ri and a frequent source of diagnostic uncertainty requiring invasive evaluation [8–11], initially directed the workup toward low‐grade glioma. The diagnostic turning point was 7 T MRI, which revealed numerous cortical microhemorrhages occult on 1.5 T imaging, shifting suspicion toward CAA‐related pathology and prompting the biopsy that confirmed the diagnosis. This is consistent with prior work demonstrating that 7 T MRI substantially improves detection of cerebral microbleeds through enhanced susceptibility effects and spatial resolution [12–14]. It also reproduces an observation reported by Ozutemiz et al. [15] in whose biopsy‐proven case the Boston criteria were unmet at both 1.5 and satisfied only at 7 T. We note that the criteria are not validated above 3 T and do not address the dependence of their hemorrhagic element on acquisition parameters. Because cortical microhemorrhages are a key imaging feature supporting the diagnosis of CAA and its inflammatory variant, higher‐field‐strength MRI may resolve diagnostic uncertainty where conventional imaging fails to explain the clinical presentation, although access to ultra‐high‐field MRI remains limited.

This dependence matters beyond the diagnosis of CAA‐ri. Eligibility for antiamyloid monoclonal antibody therapy is governed by fixed hemorrhagic thresholds counted on conventional MRI, with exclusion at five or more microhemorrhages or any cortical superficial siderosis [16], and the inflammatory response to vascular amyloid seen in CAA‐ri is mechanistically continuous with amyloid‐related imaging abnormalities. Case 1 had biopsy confirmed vascular amyloid yet would have counted zero microhemorrhages on the imaging she received. How such thresholds should be interpreted when detection varied with field strength and sequence remains unresolved.

The clinical presentation in Case 1, which included lethargy, cognitive dysfunction, and seizures, was consistent with previously reported manifestations of CAA‐ri [10, 17, 18]. Corticosteroid therapy is the standard treatment and is often associated with rapid clinical and radiographic improvement [19–21]. However, despite declining a full corticosteroid regimen, this patient achieved gradual stabilization and near‐complete radiographic resolution over several months. Spontaneous or partial recovery has been described in a minority of cases and may reflect intrinsic immune‐regulatory mechanisms [17, 19], suggesting the inflammatory process can occasionally follow a self‐limited trajectory [20].

Case 2 illustrates the diagnostic complexity of CAA‐ri in elderly patients with multiple comorbidities. Progressive cognitive decline, severe hypertension, and underlying small‐vessel ischemic disease initially suggested hypertensive encephalopathy or worsening vascular dementia, and the inflammatory component could easily have been misattributed to pre‐existing neurodegenerative or vascular pathology. The imaging feature that should prompt consideration of CAA‐ri is vasogenic edema, seen as T2/FLAIR hyperintensity or hypodensity on CT, in regions that also harbor microhemorrhages on GRE or susceptibility‐weighted imaging. Pure CAA typically does not cause edema; spatially associated edema suggests active inflammation and blood‐brain barrier breakdown driven by an immune response to amyloid‐*β*. Even in the absence of prior imaging, this pattern should guide the clinician toward immunosuppressive treatment rather than expectant management alone.

Early recognition of the inflammatory process in Case 2 allowed prompt initiation of high‐dose corticosteroids, with rapid improvement in attention and alertness and radiographic resolution of vasogenic edema, consistent with prior reports [19, 21, 22]. Her subsequent cognitive decline likely reflects underlying dementia related to chronic vascular disease and possible Alzheimer pathology, conditions frequently associated with cerebral amyloid angiopathy. Together, these cases demonstrate that while corticosteroid therapy often produces rapid symptomatic improvement, long‐term neurological outcome may be shaped by coexisting neurodegenerative or vascular disease, and that variability in diagnostic timing, imaging modality, and therapeutic decisions can significantly influence clinical management.

Our report has several limitations, most notably the anecdotal nature of case reports and the loss of one patient to follow‐up. Both patients had multiple chronic conditions that may have influenced cognition and imaging independently of CAA‐ri. Furthermore, the observational design precludes definitive conclusions about causality, as the spontaneous resolution in one patient cannot be clearly attributed to treatment or the natural course of the disease.

Greater awareness of this entity and a systematic diagnostic approach incorporating advanced neuroimaging may facilitate earlier recognition and treatment. Future studies are needed to define the optimal diagnostic strategy, clarify the role of emerging imaging modalities, and establish consensus guidelines for the diagnosis and management of this complex and potentially reversible condition.

## Funding

The authors received no financial support for the research, authorship, and/or publication of this article.

## Ethics Statement

Ethical approval was not required for this case report in accordance with institutional policy, as it describes the retrospective clinical course of a single patient at each hospital and does not constitute research involving human subjects.

## Consent

Informed consent was obtained from the patients for the publication of this case report and any accompanying images.

## Conflicts of Interest

The authors declare have no conflicts of interest.

## Supporting Information

Additional supporting information can be found online in the Supporting Information section.

## Supporting information


**Supporting Information** CARE checklist of information to include when writing a case report.

## Data Availability

Data are available upon request for the journal to review.
