# Supplementary material for: Diagnostic and Therapeutic Nuances in Cerebral Amyloid Angiopathy–Related Inflammation: Case Reports and Literature Review
Source: Case Rep Neurol Med. 2026 Nov 15;2026:9463046. doi: 10.1155/crnm/9463046 (PMC13770271; doi:10.1155/crnm/9463046)
Supplement: Supplementary file 1 [file CRNM-2026-9463046-s001.docx]

**CARE Checklist of information to include when writing a case report**

| **Topic** | **Item No** | **Checklist item description** | **Reported on Page Number/Line Number** | **Reported on Section/Paragraph** |
| --- | --- | --- | --- | --- |
| Title | 1 | The diagnosis or intervention of primary focus followed by the words “case report” | 1 |  |
| Key Words | 2 | 2 to 5 key words that identify diagnoses or interventions in this case report, including "case report" | 41 |  |
| Abstract  (Structured summary) | 3a | Background: state what is known and unknown; why the case report is unique and what it adds to existing literature. | 18 |  |
|  | 3b | Case Description: describe the patient’s demographic details, main symptoms, history, important clinical findings, the main diagnosis, interventions, outcomes and follow-ups. | 20-30 |  |
|  | 3c | Conclusions: summarize the main take-away lesson, clinical impact and potential implications. | 31-36 |  |
| Introduction | 4 | One or two paragraphs summarizing why this case is unique **(may include references)** | 71-80 |  |
| Patient Information | 5a | De-identified patient specific information | 85 |  |
|  | 5b | Primary concerns and symptoms of the patient | 87 |  |
|  | 5c | Medical, family, and psycho-social history including relevant genetic information | 86-87 |  |
|  | 5d | Relevant past interventions with outcomes | N/A |  |
| Clinical Findings | 6 | Describe significant physical examination (PE) and important clinical findings | 89-96 |  |
| Timeline | 7 | Historical and current information from this episode of care organized as a timeline | 89-116 |  |
| Diagnostic Assessment | 8a | Diagnostic testing (such as PE, laboratory testing, imaging, surveys). | 89-104 |  |
|  | 8b | Diagnostic challenges (such as access to testing, financial, or cultural) | 100 |  |
|  | 8c | Diagnosis (including other diagnoses considered) | 214 |  |
|  | 8d | Prognosis (such as staging in oncology) where applicable | N/A |  |
| Therapeutic Intervention | 9a | Types of therapeutic intervention (such as pharmacologic, surgical, preventive, self-care) | 113 |  |
|  | 9b | Administration of therapeutic intervention (such as dosage, strength, duration) | 113 |  |
|  | 9c | Changes in therapeutic intervention (with rationale) | 113 |  |

| Follow-up and Outcomes | 10a | Clinician and patient-assessed outcomes (if available) | 115 |  |
| --- | --- | --- | --- | --- |
|  | 10b | Important follow-up diagnostic and other test results | 116 |  |
|  | 10c | Intervention adherence and tolerability (How was this assessed?) | 116 |  |
|  | 10d | Adverse and unanticipated events | N/A |  |
| Discussion | 11a | A scientific discussion of the strengths AND limitations associated with this case report | 243 |  |
|  | 11b | Discussion of the relevant medical literature **with references** | 173 |  |
|  | 11c | The scientific rationale for any conclusions (including assessment of possible causes) | 237 |  |
|  | 11d | The primary “take-away” lessons of this case report (without references) in a one paragraph conclusion | 258 |  |
| Patient Perspective | 12 | The patient should share their perspective in one to two paragraphs on the treatment(s) they received | 116 |  |
| Informed Consent | 13 | Did the patient give informed consent? Please provide if requested | **Yes X** | **No** |

Please leave this space alone as it will be supplemented by the editorial office when needed.
